# Effervescent Tablet Preparation by Twin-Screw Melt Granulation with Sorbitol as a Melt Binder †

**DOI:** 10.3390/pharmaceutics17050676

**Published:** 2025-05-21

**Authors:** Zoltán Márk Horváth, Kirils Kukuls, Alīna Jaroslava Frolova, Marta Žogota, Elżbieta Maria Buczkowska, Līga Pētersone, Valentyn Mohylyuk

**Affiliations:** Leading Research Group, Faculty of Pharmacy, Rīga Stradiņš University, 21 Konsula St., LV-1007 Riga, Latvia

**Keywords:** granulation, melt granulation, twin-screw melt granulation, effervescent granules, effervescent tablets, sorbitol, polyols

## Abstract

**Methods**: Effervescent granules containing citric acid and sodium bicarbonate were successfully prepared for the first time via TS-MG using a polyol (sorbitol) as a melt binder. **Results**: Processing parameters, specifically granulation temperature and screw speed, were systematically varied to investigate their influence. The granulation efficiency, inversely related to the wt.% of fines, decreased in the following order across the tested conditions (granulation temperature–screw speed; ℃-rpm): 95-6 > 100-5 > 90-5 > 100-7 > 90-7. Granulation temperature had a minimal impact on the bulk and tapped densities of the uncalibrated granules, whereas increased screw speed led to higher densities, associated with a reduced proportion of fines. The tensile strength of the resulting effervescent tablets increased with granulation temperature and was generally higher for tablets derived from granules with higher granulation efficiency. The residence time within the TS-MG barrel decreased with increasing temperature and screw speed. Notably, the greatest effect of granulation temperature on tensile strength occurred between 90 and 95 °C, particularly under longer residence times. The disintegration time of the tablets was shortest for the 90 °C and 5 rpm condition, corresponding to the lowest tensile strength, while tablets across formulations showed consistent homogeneity as indicated by similar pH values post-disintegration. **Conclusions**: These findings underscore sorbitol’s suitability as a melt binder and highlight the interplay between TS-MG parameters and the physical characteristics of effervescent granules and tablets.

## 1. Introduction

In 1767, the English chemist Joseph Priestley discovered how to carbonate water by dissolving carbon dioxide in it, laying the foundation for effervescent beverages and later medicinal uses [1]. This led to the development of effervescent tablets, which became common in the 19th century. Effervescent formulations contain acids or acid salts (e.g., citric acid) and carbonates or bicarbonates (e.g., sodium bicarbonate) that release carbon dioxide gas when dissolved in water through a neutralisation reaction. The resulting fizzing improves the taste, allows faster dissolution and absorption, and makes swallowing easier [2]. However, they require special packaging to prevent premature reactions and are more complex and costly to manufacture [2].

The preparation of effervescent granules can be achieved via dry granulation using roller compaction [3,4], wet granulation [5,6,7], or melt granulation [8,9,10] with high-shear mixer–granulator, fluid bed granulator, and hot melt extruder methods. Wet granulation involves the addition of a liquid solvent into the dry powder mixture [11]. The solvent method has disadvantages and limitations. It requires a time-/resource-consuming drying step. Since water-containing solvents as components of binding solutions initiate the neutralisation reaction, they are inapplicable for effervescent formulations [12]. The final moisture content of effervescent granules is also a critical parameter [13]. While this makes dry granulation a better option for the preparation of effervescent granules since it does not require the use of a binding solution and thus the additional drying step, it has its own disadvantages such as the necessity of a relatively higher weight fraction of excipients in order to obtain desirable granule and tablet mechanical properties [14,15,16,17].

Wet granulation with an organic solvent-based binding solution is the most commonly applied method for effervescent granulation [2], which leaves other methods such as melt granulation under-investigated. The major benefits of the melt granulation method for the preparation of effervescent granules includes the elimination of the need for liquid solvents, which is required in wet granulation sometimes with a residual amount of water, in which case melt granulation reduces the risk of premature neutralisation reaction before the product is used [12]. Additional benefits of melt granulation are that there is no need for a drying step, less equipment and time is required, and it is economically preferable [18]. Melt granulation is simpler than the traditionally used wet granulation method for the manufacturing of effervescent granules due to the avoidance of the use of binding solutions and the reduction of operations during the manufacturing process, reducing the number of critical parameters. In accordance with the Manufacturing Classification System (MCS), process simplification and the reduction of critical process parameters decrease the risk of deviation in target quality parameters and reduce the quality control load [19].

The temperature used during melt granulation is usually defined by the melting temperature range of the melt binder. The thermal stability of the drug and/or excipients sets the upper limit for processing temperature [14]. Based on this, APIs and excipients can be classified into four categories in the TS-MG formulation system, according to two factors: the thermal/chemical stability of the API (poor or good) and the drug–binder miscibility (poor or strong) [20]. Melt granulation can be carried out by various equipment, such as a fluidised bed processor [8,10], high-shear mixer [10], and twin-screw melt granulator (TS-MG) [9]. However, a major advantage of TS-MG includes it being a continuous process with controlled temperature and shear history, which is one of the main drivers behind the pharmaceutical industry’s interest in TS-MG due to the list of advantages continuous manufacturing offers over batch manufacturing [14]. Furthermore, TS-MG allows for the precise tracking of the time the components spend inside the TS-MG barrel (residence time) at the specified processing parameters with the introduction of a small amount of dye at the feed port [21].

The selection of an appropriate melt binder for melt granulation is crucial, especially for the preparation of effervescent granules due to the effervescent ingredients that have low degradation temperatures, such as citric acid (160–270 °C) and sodium bicarbonate (100–180 °C) [22]; a melt binder with a melting temperature range below 100 °C is recommended. Additionally, other properties of the melt binder, such as the following rheological properties; melt viscosity and degradation, solidification and crystallisation temperatures, influence the granulation process and affect the properties of the resultant effervescent granules and their respective effervescent tablets [23,24,25]. While polymers (such as polyvinylpyrrolidone (PVP) or its copolymers such as polyvinylpyrrolidone-vinyl acetate (PVPVA), poly (ethylene-co-vinyl acetate), various grades of polyethylene glycol (PEG), cellulose-esters and cellulose-acrylates, polyethylene oxides (PEOs) of varying molecular weights, poly-methacrylate derivatives, and poloxamers [26]) have been widely used as melt binders, sugar alcohols (polyols) possessing comparable processing temperatures (based on their melting temperature) offer advantages regarding tablet disintegration and dissolution, as well as tablet hardness. While polyols have been widely used in pharmaceutical formulations including for hot melt extrusion (HME) in oral dosage forms [27] such as erythritol [28,29], sorbitol [29,30], mannitol [31,32], and xylitol [29,31,32], they have been under-investigated for use in TS-MG, such as the co-processing of hydroxypropylcellulose with mannitol [33], and especially lacking in research regarding their application for effervescent granulation via TS-MG [34].

The aims of this work were to investigate the effect of sorbitol as a melt binder on the granulation efficiency and tablet properties (such as tensile strength, disintegration time, and homogeneity) as a function of screw speed and granulation temperature.

## 2. Materials and Methods

Citric acid (Merck KGaA, Darmstadt, Germany) and sodium bicarbonate (Sisecam Soda Lukavac d.o.o., Lukavac, Bosnia and Herzegovina) were used as the effervescent ingredients. The polyols xylitol (Sigma; Thermo Fisher Scientific, Vantaa, Finland), sorbitol (Parteck^®^ SI 200, Merck KGaA, Darmstadt, Germany), isomalt (galenIQ™ 721; Beneo-Palatinit GmbH, Südzucker AG, Obrigheim, Germany), mannitol (Pearlitol^®^ 160C; Roquette Frères, Beinheim, France), and sucrose (Nordzucker AG, Kantvik, Finland) were considered as melt binder candidates. Sodium stearyl fumarate (PRUV^®^; JRS Pharma, Germany) was introduced as a water-soluble lubricant for the tableting of granulated formulations. Copper (II) oxide (particles less than 10 µm) was used as a dye to track the residence time of particles inside the TS-MG barrel.

### 2.1. Differential Scanning Calorimetry (DSC)

**Figure 1:**
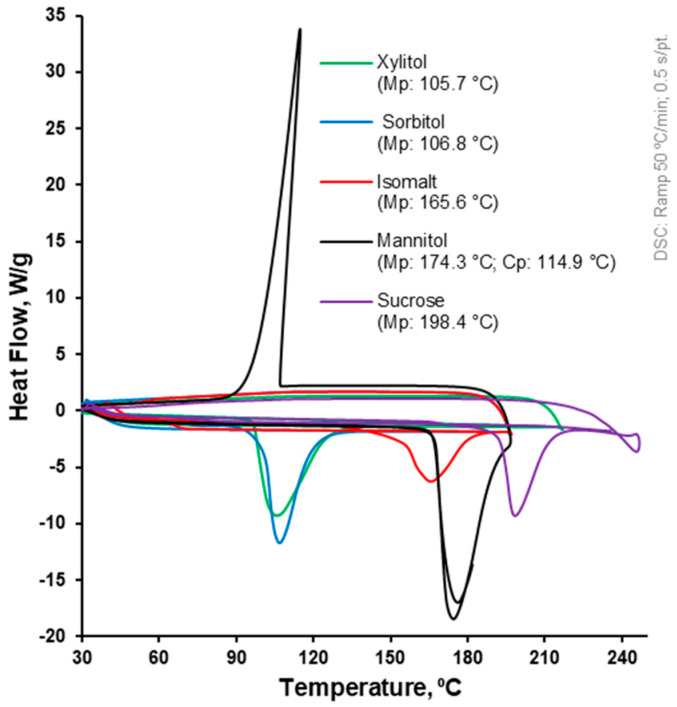
The DSC profiles of polyols (xylitol, sorbitol, isomalt, mannitol, and sucrose) at a heating rate of 50 °C/min and continuous nitrogen purge at 50 mL/min.

To investigate the thermal properties of different polyols as melt binder candidates, a heat-flux DSC (DSC Q20; TA Instruments, New Castle, DE, USA) was conducted to characterise their thermal behaviour (Figure 1). The samples were weighed (5–8 mg) into aluminium DSC pans and heated from 30 °C to 200 or 250 °C, then cooled to 30 °C and heated again to 200 or 250 °C with a heating/cooling rate of 50 °C/min and a continuous purge of nitrogen gas at 50 mL/min. The data were processed with a Universal V4.5A software (TA Instruments, New Castle, DE, USA), and the peak melting and peak crystallisation temperatures were determined [35].

### 2.2. Sample Preparation

The citric acid and sodium bicarbonate were ground separately using a pestle and mortar, and sieved through a 200 µm mesh. Citric acid, sodium bicarbonate, and sorbitol with a maximum particle size of 200 µm were kept in a desiccator for a week to demoisturise. The citric acid and the sodium bicarbonate were then mixed in a 1:3 molar ratio. Sorbitol was then added to the powder mix, making up 30 wt.%. The powder mix was kept in a desiccator at all times before introducing the mix into the TS-MG barrel. The produced effervescent granules of all formulations were collected from the conveyor belt and immediately stored in a desiccator at all times outside of analysing the granules for their properties to minimise the absorption of moisture from the atmosphere.

### 2.3. Moisture Content Measurement

The moisture content of the dry powder mix was measured with a Moisture Analyzer (HX204; Mettler Toledo GmbH, Greifensee, Switzerland) at 105 °C with a switch-off criterion of 0.01%/300 s. The measurement was carried out in triplicate.

### 2.4. Optical Microscopy

An optical microscope (BA410E; Motic, Xiamen, China) was used to visually observe the ground citric acid and sodium bicarbonate and the sorbitol. The microscope was equipped with a 50 W halogen lamp and a Motic EC-H Plan 10×/0.25 objective lens. The images were collected by a Moticam ProS5 Lite camera controlled by the Motic Images Plus 3.0 software (Motic, Xiamen, China). Blue illumination was applied to the surface of the samples for improved image clarity [36].

### 2.5. Twin-Screw Melt Granulation

**Figure 2:**
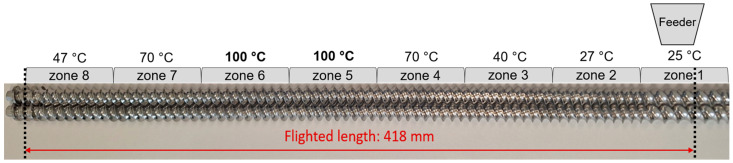
Screw design for TS-MG (exemplified by processing temperatures for formulations 100-5 and 100-7).

Twin-screw melt granulation was carried out using a Pharma 11 Extruder without a nozzle, a volumetric feeder, and a conveyor belt (Thermo Electron Corporation, Karlsruhe, Germany). The barrel that was used had a flighted length of 418 mm and a diameter of 11 mm with a length/diameter ratio (L/D) of 38:1. The screw design consisted of 2 L/D long helix feed screw elements at the feed port (zone 1), followed by 1 L/D helix feed screw elements, and a 1 $\frac{1}{2}$ L/D discharge element at the end. A total of 10 mm protruded from the barrel of which 5 mm was part of the discharge element. The barrel had eight heating zones (Figure 2) with zone 1 fixed at room temperature (25 °C), and the temperature of each zone was set according to the required maximum granulation temperature (Table S1) [14].

Considering the melting range of sorbitol and the degradation temperature of the effervescent ingredients, the temperature range of 90 to 100 °C was investigated at low screw speeds of 5 to 7 rpm to more easily understand the impact of the processing parameters. Therefore, five formulations (100-5, 100-7, 90-5, 90-7, and 95-6) with 95-6 in triplicate (cp1, cp2, and cp3) for method reproducibility were prepared via TS-MG where the first number represents the maximum granulation temperature used in degrees Celsius, the second number after the hyphen represents the screw speed in terms of rpm, and cp represents the “centre point”. The dry powder mixture was introduced into the barrel at a feed rate of 1.4 g/min, passed through the barrel from zone 1 to zone 8 at respective temperature and screw speed (Table S1) and was allowed to free-fall onto the conveyor. The conveyor, with a length of 587 mm, moved at a speed of 100 mm/s. The samples were allowed to free-fall at the end of the conveyor into labelled containers. The residence time of the powder mix particles inside the TS-MG barrel was determined for each formulation (Table S2) by adding a dye (copper (II) oxide, tip of spatula) as a pulse into the powder feed port, and visually checking the colour intensity at the outlet [21]. The time taken for the first appearance of the dye at the outlet was taken as the residence time.

### 2.6. Calculation of TS-MG Free Volume and Fill Level

The free volume fill level % was calculated for each formulation (Table S2) based on the feed rate, the residence time, and the extruder geometry with stainless steel screw elements (Chronifer M15KL; Thermo Fisher Scientific, Karlsruhe, Germany). The volume of the barrel was obtained by multiplying the cross-section area of the barrel (182.82 mm^2^) with the flighted length (418 mm). The volume of the screw shaft was obtained by multiplying the cross-section area of the shaft (18.81 mm^2^) with the flighted length (418 mm). The volume of each type of screw element was obtained by dividing the average weight of each type of screw element (long helix feed screw: 4958.89 mg, helix feed screw: 2465.91 mg, discharge element: 4285.64 mg) by the reported density of the screw elements (7.7 g/cm^3^ [37]). The free volume was calculated in 2.75 mm segments per screw element type, and their sum over the flighted length of 418 mm resulted the total free volume (36,241.51 mm^3^).

The weight of the material inside the barrel was then calculated for each formulation by multiplying the residence time with the feed rate. The weight of the material inside the barrel was divided by the calculated true density of the mixture (1.814 mg/mm^3^) to obtain the volume of material inside the barrel. The calculated true density of the composition (1.8246 g/cm^3^) was obtained based on the true density (*ρ_t_*) of citric acid (1.665 g/cm^3^) [38], sodium bicarbonate (2.2 g/cm^3^) [39], sorbitol SI 200 (1.489 g/cm^3^) [40], and their shares (x, *w*/*w*) using the additive methodology and the following equation [41]:

$$\rho_{t}=\left(\rho_{exc\;1}\cdot x_{exc\;1}\right)+\ldots +\left(\rho_{exc\;i}\cdot x_{exc\;i}\right)$$

The free volume occupied by the mixture was then calculated for each formulation by dividing the volume of material inside the barrel by the total free volume, and converted into percentages.

### 2.7. Cooling Rate of Granules

The cooling rate of the effervescent granules was measured using a FLIR ONE^®^ Pro LT (FLIR Systems, Inc., assembled in China, designed in Santa Barbara, CA, USA) smartphone accessory thermal imaging camera (third generation) [42]. The camera was fixed with a minimum observation distance of 20 cm between the surface of the granules and the camera lens to ensure focus. The temperature of five separate effervescent granules was measured over a time period of one minute.

### 2.8. Sieve Analysis of Effervescent Granules

The sieving of the effervescent granules was carried out using a vibratory sieve shaker (AS 200 digit cA; Retsch GmbH, Haan, Germany) with the following mesh: 2 mm, 1 mm, 800 µm, 500 µm, 400 µm, and 200 µm. The equipment operated at an amplitude of 1.00 mm for 5 min with an interval after every 10 s. The weight fractions obtained in grams were converted to percentages and cumulative weight fraction graphs were plotted. The granulation efficiency was investigated based on the wt.% of fines produced per formulation as a function of screw speed and maximum granulation temperature.

### 2.9. Bulk and Tapped Density Measurement

The bulk and tapped density of the effervescent granules were measured using a tapped density tester (SVM II; Erweka GmbH, Langen, Germany). The measurements were carried out using a 250 mL cylinder. Measurements were taken before any taps, after 10, 110, 1110, and 3110 taps. The equipment operated with a speed of 250 taps/min and a tapping height of 3 ± 0.2 mm. The measurements were carried out in triplicate for each sample and the averaged values were used for calculations [14].

### 2.10. Preparation of Effervescent Tablets

Prior to tableting, the effervescent granules of each formulation were size-calibrated through a 1.6 mm mesh. To each formulation, 1.5 wt.% of sodium stearyl fumarate that had previously been sieved through a 0.5 mm mesh was added and mixed for three minutes. The mixtures were then once again sieved through a 1.6 mm mesh. The formulations were tableted with 11.28 mm flat punches to obtain a target mass of approximately 350 mg using the compaction simulator STYL’One Nano (Medelpharm, Beynost, France). The compression cycles simulated a V-shape, and the tableting speed simulated 90 mm/s, with a compaction force of 20 kN (equivalent to 200 MPa at the used punch diameter of 11.28). The powder feeding into the die was performed automatically with the feed shoe.

### 2.11. Tablet Hardness Measurement and Tensile Strength Calculation

The tablet thickness (t) and diameter (d), as well as tablet hardness (breaking force, F) were measured (n = 10) by a tablet tester (ST50 WTDH; SOTAX AG, Aesch, Switzerland) immediately after the compaction. The tensile strength (τ, MPa) was calculated by employing the following equation:

$$\tau =\frac{2F}{\pi dt}$$

### 2.12. Tablet Disintegration Measurements

The disintegration test of the effervescent tablets was carried out in vitro using 100 mL of purified water. A timer was started as soon as the tablet immersed within the aqueous solution until the tablet fell apart and vigorous bubbling was no longer observed. The disintegration test was carried out for six tablets per formulation, and the average values with RSD were obtained [43].

### 2.13. Tablet Homogeneity Measurements

The homogeneity of the effervescent tablets was compared by measuring the pH of the aqueous solutions used for the disintegration test directly after full dissolution of the tablets with a pH meter (SevenDirect SD23, Mettler Toledo GmbH, Greifensee, Switzerland).

### 2.14. Statistical Analysis

A one-way analysis of variance (ANOVA) test was used to compare the means of the disintegration time of the effervescent tablets for the formulations using the built-in possibilities of the current version of Excel (Microsoft 365; Redmond, Washington, DC, USA).

In an attempt to investigate the effects of the factors (granulation temperature, screw speed, residence time) on the responses (fines wt.%, tensile strength, disintegration time) and explore the optimisation possibilities of the factors to obtain the desired responses, a statistical software tool (MODDE 13.1 Pro; Sartorius, Malmö, Sweden) used for Design of Experiments (DoE) was employed. For this purpose, a full factorial design with an interaction model and three centre points was selected for the initial screening.

## 3. Results and Discussion

Twin-screw melt granulation appears to be a promising method for the preparation of effervescent granules using citric acid and sodium bicarbonate, and sorbitol was selected as an appropriate melt binder from the various polyols that were investigated regarding their thermal properties via DSC heat–cool–heat measurements (Figure 1).

During the first heating cycle of the DSC measurements, the peak melting temperature of the polyols increased from xylitol (105.7 °C) to sorbitol (106.8 °C) to isomalt (165.6 °C) to mannitol (174.3 °C) to sucrose (198.4 °C). During the cooling cycle, mannitol had a crystallisation temperature of 114.9 °C, the same melting onset, and approximately the same peak melting temperature of 176.1 °C (vs. 174.3 °C) during the second heating cycle. Considering the broad but low degradation temperature ranges of citric acid (160–270 °C) and sodium bicarbonate (100–180 °C) [22], sorbitol with one of the lowest peak melting temperatures (106.8 °C) and the lowest onset melting temperature was selected as an appropriate melt binder for the purpose of this work.

**Figure 3:**
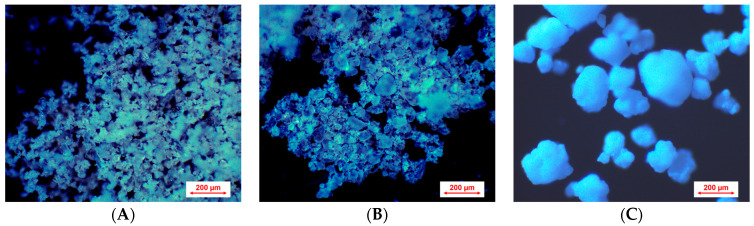
Optical microscopy images of (**A**) ground citric acid, (**B**) ground sodium bicarbonate, and (**C**) sorbitol at a magnification of ×10.

Ground and sieved (<200 µm) citric acid and sodium bicarbonate, along with sorbitol, were visually observed with an optical microscope (Figure 3). While the particles of citric acid and sodium bicarbonate particles appeared irregular in shape, the sorbitol particles appeared spherical. The number average of the citric acid and sodium bicarbonate particles appeared to be below 100 µm. The particle size distribution of sorbitol (D_10_ = 90.5 ± 2.2, D_50_ = 209.3 ± 6.6, D_90_ = 405 ± 20.6) was investigated previously [44].

The dried citric acid, sodium bicarbonate, and sorbitol with a moisture content of less than 0.5% were mixed according to the sample preparation, and granulated with specific processing parameters (Table S1) and screw designs (Figure 2).

**Figure 4:**
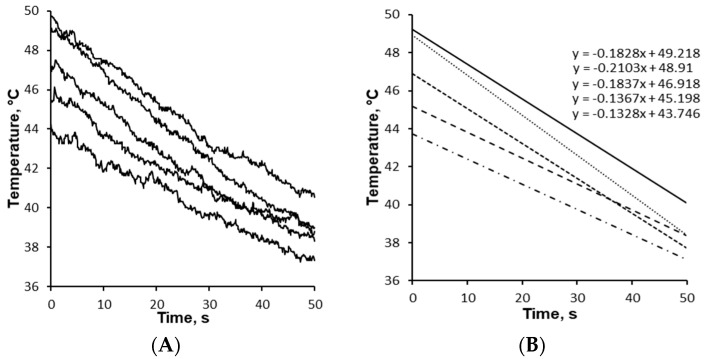
The cooling rate of the effervescent granules after free-falling from the extruder’s exit onto the conveyor belt presented as (**A**) raw data and (**B**) trendlines of raw data.

The effervescent granules exiting from the TS-MG barrel had an approximate temperature of 50 °C. Since the cooling rate/solidification of the granules affect the granule formation and properties, the cooling rate was investigated (Figure 4). The slight variation of the starting temperature of the granules was due to the different time it took for the granules to fall onto the conveyor belt (start of measurement) from the protruding screws. The cooling rate of the effervescent granules between a temperature range of 50 and 37 °C was calculated as 10.2 °C/min from an average of five measurements.

**Figure 5:**
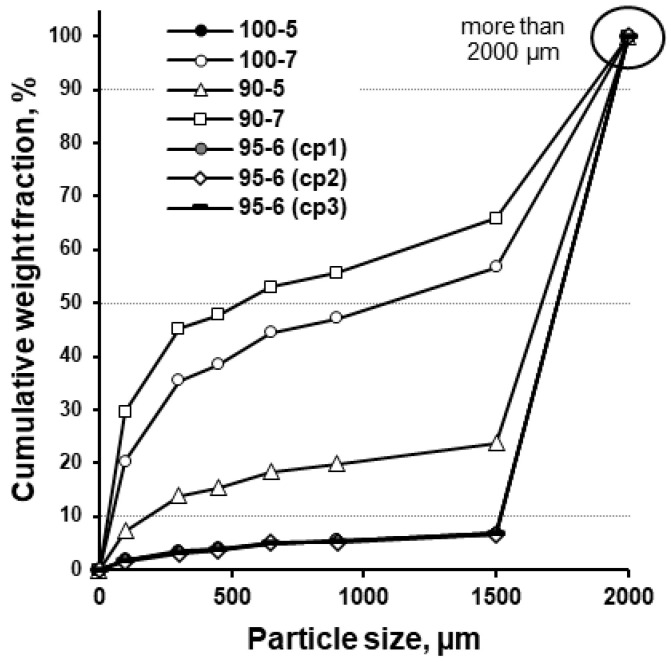
Cumulative weight fraction of twin-screw melt granulated formulations.

**Figure 6:**
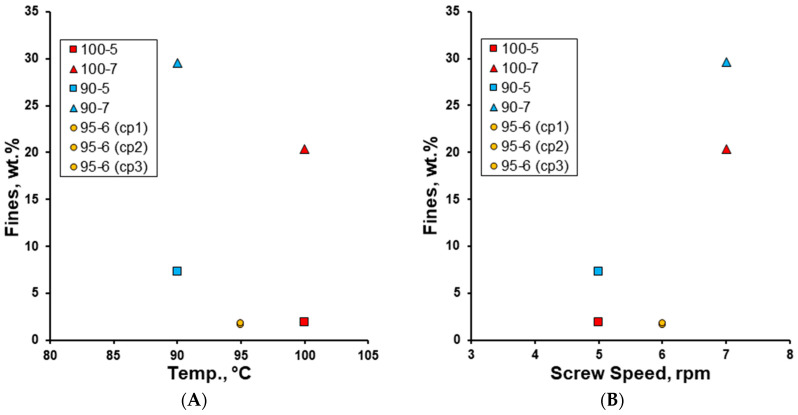
The effect of (**A**) granulation temperature (90–100 °C) and (**B**) screw speed (rpm) on the wt.% of fines (<200 µm) of the effervescent granules.

The cumulative weight fraction profiles of the effervescent granules showed a clear effect of the applied processing parameters (Figure 5). The granules prepared at a higher screw speed (7 rpm) yielded a higher percentage of granules less than 1500 µm compared to lower screw speeds. Although the abundance of granules less than 1500 µm is greater at a screw speed of 5 than 6, the difference may be explained by the different granulation temperature. Since the upper end of the size range of the granules formed exceeded 2000 µm, the D_10_ and D_50_ values were unable to be compared with the available set of sieves. Therefore, the granulation efficiency of different processing parameters was compared in terms of the fines (wt.% of material less than 200 µm). The size below 200 µm was related to non-granulated or poorly granulated material (Figure 6) because of the maximum particle size of the components (Figure 3).

The initial screening models obtained through MODDE 13.1 Pro had low coefficient of determination (R^2^) and predictive performance (Q ^2^) values (i.e., 0.55 and 0.36, 0.29 and 0.05, and 0.89 and 0.46, respectively) with low model validity (lower than 0.25) for one of the models. Despite the high reproducibility of the centre points (95-6 cp1, cp2, and cp3; Tables S1 and S2) for each model (greater than 0.83), the application of the software was unsuccessful. Therefore, each variable processing parameter was plotted against each investigated response separately.

As the granulation temperature increased, the wt.% of fines decreased. In contrast, as the screw speed increased, the wt.% of fines increased. The granulation at the mid-point of both parameters produced the lowest wt.% of fines (Figure 6).

**Figure 7:**
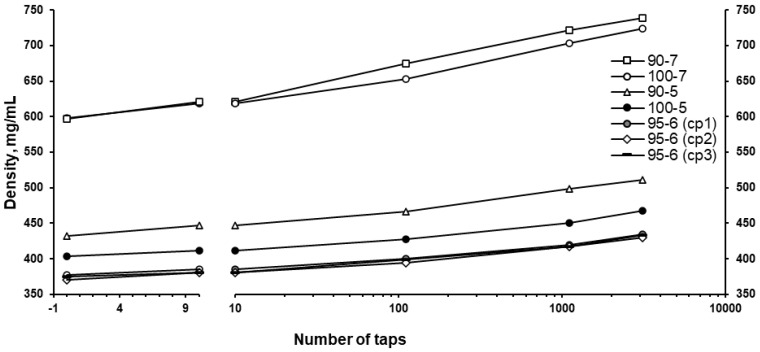
Densification kinetics of the effervescent granules (n = 3; RSD ≤ 2%).

**Figure 8:**
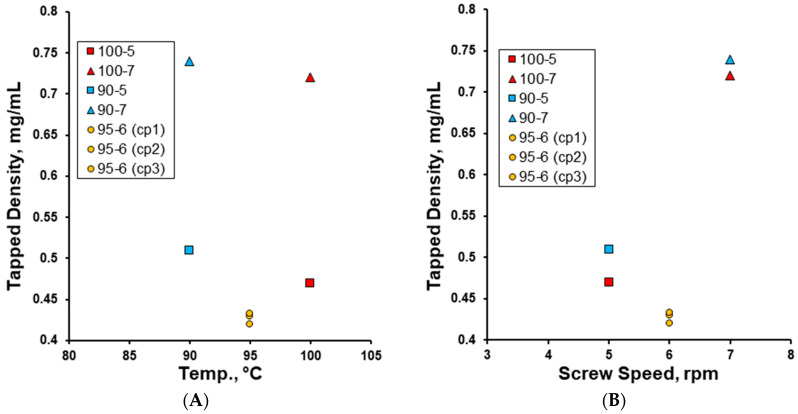
The effect of (**A**) granulation temperature (90–100 °C) and (**B**) screw speed (rpm) on the tapped density (mg/mL) of the effervescent granules.

The densification kinetics of the granules as obtained (uncalibrated) showed that while the granulation temperature had little effect on the bulk and tapped densities of the produced effervescent granules (Figure 7), an increase in the screw speed from 5 to 7 rpm resulted in granules with distinctively higher bulk and tapped densities as expected from the wt.% of fines produced at respective screw speeds (Figure 6B). With an increase in granulation temperature from 90 to 100 °C, the tapped density of the produced effervescent granules slightly decreased, whereas when the screw speed was increased from 5 to 7 rpm, the tapped density of the produced effervescent granules was distinctively higher (Figure 8). Interestingly, the granulation at the mid-point of both parameters produced granules with the lowest bulk and tapped densities. The ranges of bulk and tapped density values of the effervescent granules prepared in this work were comparable to effervescent granules prepared by other methods such as wet and dry granulation [7,45,46].

**Figure 9:**
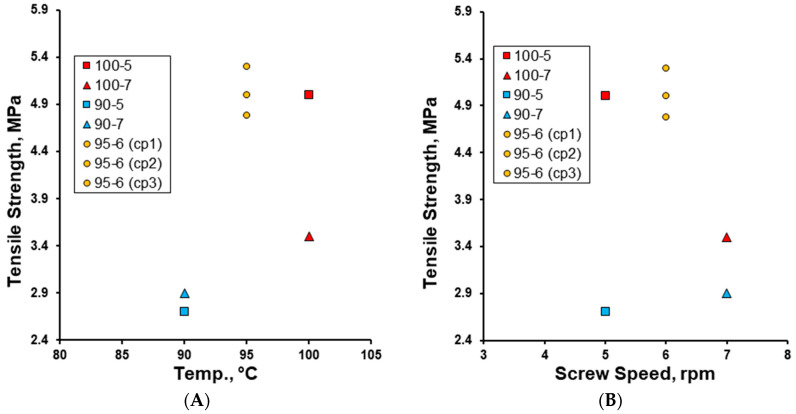
The effect of (**A**) granulation temperature (90–100 °C) and (**B**) screw speed (rpm) on the tensile strength (MPa) of the effervescent tablets.

After size-calibrating the effervescent granules through a 1.6 mm mesh and tableting thereafter, the tensile strength of the effervescent tablets (prepared at a compression force of 20 kN) was measured, and it was shown to increase with an increase in granulation temperature from 90 to 100 °C (Figure 9A). However, while the tensile strength was very close at screw speeds of 5 and 7 rpm at a granulation temperature of 90 °C, a decrease in tensile strength was observed along with the increase in screw speed at a granulation temperature of 100 °C (Figure 9B). The tensile strengths of the effervescent tablets prepared at the mid-point of both parameters were close to that at a processing parameter of 100 °C at 5 rpm. These findings confirm that the low density of the granules of formulations 95-6 and 100-5 (Figure 7) contribute to their high tensile strength, and this is in line with the results of other studies [11,16,17]. The materials with a lower density have a higher porosity (and as a consequence, a lower solid fraction) and exhibit higher fragility which exposes them to higher brittle deformation [11,16]. As a consequence of this, particles have a larger bonding area and interparticulate bonding strength, which results in a higher tensile strength for the tablets [47]. Additionally, all effervescent tablets prepared were above the minimum acceptance level of 2 MPa for tablet tensile strength [48] and showed superior tensile strength compared to tablets prepared with PEG 8000 via TS-MG [14], as well as magnesium carbonate effervescent tablets prepared via roller compaction [3].

**Figure 10:**
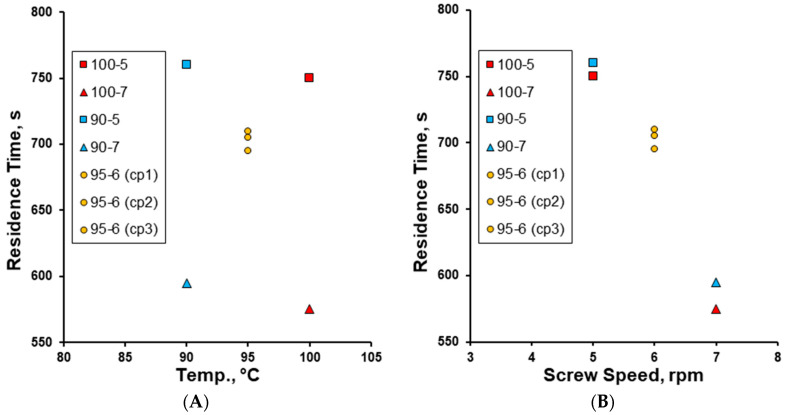
The effect of (**A**) granulation temperature (90–100 °C) and (**B**) screw speed (rpm) on the residence time (s) of the powder mix within the TS-MG barrel.

The residence time was clearly dependent on screw speed, decreasing almost linearly as the screw speed increased from 5 to 7 rpm (Figure 10). This residence time was then used to calculate the free volume fill level % for each formulation (Table S2), all of which ranged between 20 and 27%, increasing with longer residence time.

**Figure 11:**
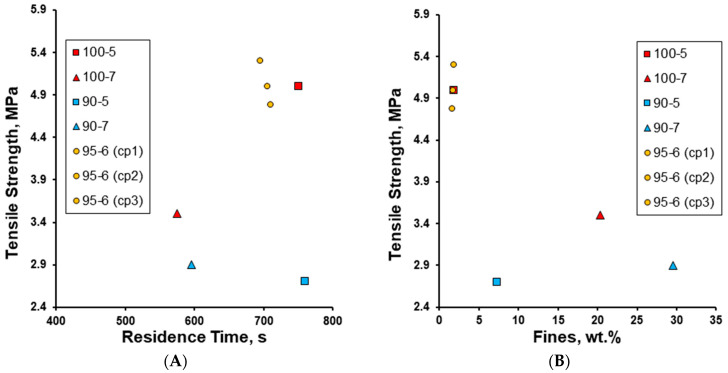
The effect of (**A**) residence time (s) of the powder mix within the TS-MG barrel and (**B**) the wt.% of fines (<200 µm) of the effervescent granules on the tensile strength (MPa) of their respective effervescent tablets.

It appears that the powder mix that spent a longer time within the TS-MG barrel due to the (lower) screw speed yielded effervescent tablets with superior tensile strengths; however, the effervescent tablet with a long residence time (760 s) but a low screw speed (5 rpm) had a much lower tensile strength (2.7 MPa), comparatively (Figure 11A). This suggests that the effect of granulation temperature on the tensile strength based on the residence time is much greater between 90 and 95 °C than between 95 and 100 °C. While a clear effect of the wt.% of fines on the tensile strength was observed—that is, the lower the wt.% of fines of the effervescent granules, the higher the tensile strength of their respective effervescent tablets—the effervescent tablet prepared from its respective granule with only 7.28 wt.% fines had a comparatively and surprisingly low tensile strength (Figure 11B).

The disintegration times, with RSD, of the effervescent tablets of formulations 100-5, 100-7, 90-5, 90-7, 95-6 cp1, 95-6 cp2, and 95-6 cp3 were measured as 77.83 ± 6.94 s, 72.83 ± 9.85 s, 62.67 ± 6.94 s, 77.83 ± 5.91 s, 72.5 ± 8.71 s, 75.6 ± 8.17 s, and 69.34 ± 9.74 s, respectively (Table S2). According to the ANOVA test, the disintegration time for the effervescent tablet prepared at a maximum granulation temperature of 90 °C and a screw speed of 5 rpm was the only one that was significantly different (*p* < 0.05) compared to the rest of the tablets. The same formulation had a distinctively different tensile strength based on the residence time and the wt.% of fines as compared to the rest of the formulations. All of the effervescent tablets exhibited acceptable disintegration times below three minutes as defined by pharmacopoeial guidelines [43,49]. The pH values of the solutions after the disintegration tests were relatively similar for all formulations in the range of 6.11–6.15 with an RSD of 0.3%; therefore, the effervescent tablets of all formulations can be considered to have good homogeneity.

Although the screw speed and the resulting residence times were lower and longer, respectively, than typical production-scale TS-MG settings, this work having focused on mechanistic investigation and granulation efficiency established as a feasibility study the potential of sorbitol as a novel melt binder in producing effervescent granules via TS-MG. Future formulation optimisation at more commercially relevant throughputs (higher feed rate and screw speed and lower residence time) may be conducted in future research. While the upper limit of the processing temperature is dictated by the thermal stability of the drug and/or excipients, with higher screw speeds and lower residence times, the amount of time the materials will be exposed to high temperatures will drastically decrease; therefore, higher processing temperatures may also be explored.

## 4. Conclusions

To the best of our knowledge, effervescent granules containing citric acid and sodium bicarbonate were successfully prepared for the first time via TS-MG using sorbitol as a melt binder. The granulation efficiency, inversely related to the wt.% of fines, decreased in the following order across the tested conditions: 95-6 > 100-5 > 90-5 > 100-7 > 90-7. Higher granulation efficiency, indicated by a lower wt.% of fines, correlated with increased tablet tensile strength. Screw speed had a stronger influence on the bulk and tapped densities than granulation temperature, due to its impact on fines content. Although tensile strength increased with temperature, the effect was more pronounced between 90 and 95 °C, especially at longer residence times. The shortest disintegration time was observed at 90 °C and 5 rpm, consistent with the tablet’s low tensile strength. Overall, the tablets displayed good homogeneity, as reflected in consistent pH values and disintegration times across formulations.

## Data Availability

The data presented in this study are available on request from the corresponding author.

## Supplementary Materials

The following supporting information can be downloaded at https://www.mdpi.com/article/10.3390/pharmaceutics17050676/s1, Table S1: TS-MG formulation and processing variables; Table S2: Physical properties of effervescent granules and tablets.
