# Supplementary material for: Effervescent Tablet Preparation by Twin-Screw Melt Granulation with Sorbitol as a Melt Binder
Source: Pharmaceutics. 2025 May 21;17(5):676. doi: 10.3390/pharmaceutics17050676 (PMC12115153; doi:10.3390/pharmaceutics17050676)
Supplement: Supplementary file 1 [file pharmaceutics-17-00676-s001.zip › pharmaceutics-3609253-supplementary.pdf]

Supplement

Table S1. TS-MG formulation and processing variables

		Formulations						
		**100-5**	**100-7**	**90-5**	**90-7**	**95-6 (cp1)**	**95-6 (cp2)**	**95-6 (cp3)**
Formulation	Citric acid wt.%				30.3			
	Sodium bicarbonate wt.%				39.7			
	Sorbitol wt.%				30			
Processing parameters	Zone 1 (°C)	25	25	25	25	25	25	25
	Zone 2 (°C)	27	27	27	27	25	25	25
	Zone 3 (°C)	40	40	40	40	40	40	40
	Zone 4 (°C)	70	70	65	65	65	65	65
	Zone 5 (°C)	100	100	90	90	95	95	95
	Zone 6 (°C)	100	100	90	90	95	95	95
	Zone 7 (°C)	70	70	65	65	65	65	65
	Zone 8 (°C)	47	47	44	44	40	40	40
	Feed rate (g/min)				1.4			
	Screw speed (rpm)	5	7	5	7	6	6	6

Torque (Nm)	3.84	4.56	5.64	4.56	6	5.52	6.36

Table S2. Physical properties of effervescent granules and tablets (Av. ± RSD)

	**100-5**	**100-7**	**90-5**	**90-7**	**95-6 (cp1)**	**95-6 (cp2)**	**95-6 (cp3)**
Residence time (s)	750	575	760	595	705	710	695
Bulk density (g/mL)	0.41	0.6	0.43	0.6	0.38	0.385	0.37
Tapped density (g/mL)	0.47	0.72	0.51	0.74	0.43	0.43	0.42
Free volume fill level (%)	26.46	20.29	26.82	21.00	24.88	25.05	24.52
**Tablets obtained at 200 MPa**							
Tablet weight (mg)	336.9 ± (1.7)	359.5 ± (1.2)	329.5 ± (4.1)	346.8 ± (1.7)	339 ± (2.7)	343.6 ± (2.7)	334 ± (2.6)
Tablet thickness (mm)	2.17 ± (1.35)	2.33 ± (1.02)	2.23 ± (2.55)	2.29 ± (1.88)	2.23 ± (1.49)	2.24 ± (1.55)	2.23 ± (1.5)
Tablet diameter (mm)	11.24 ± (7.6)	11.26 ± (0.06)	11.26 ± (0.06)	11.26 ± (0.04)	11.24 ± (0.04)	11.24 ± (0.05)	11.24 ± (0.04)
Tablet hardness (N)	191 ± (7.6)	146 ± (9)	108 ± (31.1)	118 ± (12.1)	197 ± (12.1)	187 ± (11.4)	209 ± (13.5)
Tensile strength (MPa)	5.0 ± (6.27)	3.5 ± (7.73)	2.7 ± (26.87)	2.9 ± (10.76)	5.0 ± (10.58)	4.75 ± (10.46)	5.3 ± (10.6)
Disintegration time (s)	77.83 ± (6.94)	72.83 ± (9.85)	62.67 ± (6.94)	77.83 ± (5.91)	72.5 ± (8.71)	75.6 ± (8.17)	69.34 ± (9.74)
